# The exposure of adolescent girls with/without disabilities to discrimination: cross-sectional analyses of nationally representative surveys undertaken in 37 countries

**DOI:** 10.1093/pubmed/fdag054

**Published:** 2026-06-27

**Authors:** Eric Emerson, Katrina Scior, Gwynnyth Llewellyn

**Affiliations:** Faculty of Health and Medicine, Lancaster University, Lancaster, LA1 4YW, UK; School of Health Sciences, University of Sydney, Camperdown NSW 2050, Australia; College of Nursing and Health Sciences, Flinders University, Sturt Rd, Bedford Park SA 5042, Australia; Department of Clinical, Educational & Health Psychology, University College London, Gower Street London WC1E 6BT, UK; School of Health Sciences, The University of Sydney, Camperdown NSW 2050, Australia; WHO Collaborating Centre for Strengthening Rehabilitation Capacity in Health Systems, Camperdown NSW 2050, Australia; Melbourne School of Population and Global Health, University of Melbourne, 207 Bouverie St, Carlton VIC 3053, Australia

**Keywords:** disabilities, social determinants, young people

## Abstract

**Background:**

Little is known about the prevalence of exposure to discrimination among adolescent girls with/without disability, especially in low- and middle-income countries.

**Methods:**

Secondary analysis of nationally representative data collected on self-reported discrimination from 30 705 adolescent girls in 37 low- and middle-income countries in Round 6 (2017–2023) of UNICEF’s Multiple Indicator Cluster Surveys (MICS).

**Results:**

Our results indicated that: (i) adolescent girls with disability were 1.66 times more likely to be exposed to discrimination than adolescent girls without disability; (ii) adolescent girls with disability were at higher risk of exposure to all forms of discrimination measured; (iii) they were also significantly more likely to be exposed to discrimination if they lived in poorer households and had mothers with lower than secondary level education; (iv) there was no association between country level of human development and the magnitude of the risk of adolescent girls with disability being exposed to discrimination.

**Conclusions:**

Adolescent girls with disability are at significantly increased risk of exposure to discrimination based on disability and other characteristics (e.g. age, gender). Monitoring risk of discrimination among people with disability needs to move beyond a narrow focus on disability-related discrimination.

## Introduction

The risk of discrimination against people with disability underpinned the development of disability discrimination legislation in many countries^1^ and of the 2006 United Nations Convention on the Rights of Persons with Disability, now ratified by over 190 countries.^2^ Discrimination represents a violation of the right of people to participate in society on equal terms with others and increases risk of exposure to social determinants of poorer health.^3^ That exposure to discrimination is detrimental to health,^4–11^ discrimination is an important public health issue.^3^

The 2024 United Nations *Disability and Development Report* contains data from 19 countries or areas, indicating that the percentage of persons with disability who reported facing discrimination due to their disability ranged from 1%–20%.^1^ However, the prevalence of exposure to discrimination among people with disability is likely to be higher as people with disability are also at risk of exposure to discrimination based on other personal characteristics. Indeed, some evidence suggests that working age adults with disability may be 1.5 times more likely than their non-disabled peers to be exposed to non-disability related discrimination.^12^

Knowledge about the relationship between disability and discrimination has four limitations: (i) over-reliance on studies undertaken in high-income countries; (ii) extensive use of unrepresentative convenience samples; (iii) use of measures of disability that underestimate the prevalence of disability and/or produce biased samples of people with disability; and (iv) few studies, which have focused on people with disability during adolescence.

Regarding the first two issues, two recent systematic reviews investigated the exposure of people with disability to discrimination in the workplace^13^ and by health care providers.^14^ While including data from 87 studies, only two were undertaken in low- or middle-income countries (LMICs). Both used small convenience samples. More recently a few studies have begun to redress these inequities. For example, Emerson and Llewellyn pooled representative data across 29 LMICs, reporting that women with disability aged 18–49 were 1.9 times more likely to be exposed to discrimination in the previous 12 months than women without disability.^15^

With regard to the measurement, most surveys have used the Washington Group Short Set of questions (WG-SS) to identify disability.^1^ However, data from a number of studies have suggested that the WG-SS significantly underestimates the prevalence of disability, especially among younger adults. It also introduces systematic biases by not capturing people with disability associated with mental health problems.^16–22^

Few population-based studies have focused on the association between disability and discrimination during adolescence. Analysis of the 2013 British Columbia Adolescent Health Survey indicated that exposure to any form of discrimination was significantly greater for adolescents with disabilities than adolescents with no disability.^23^ Similarly, analysis of the 2018 Health Behaviour in School-Aged Children survey undertaken in Ireland, reported that youth with disability were significantly more likely than non-marginalized youth to report discrimination due to gender (43% vs. 37%), disability (18% vs.7%), sexual orientation (14% vs. 10%), religion (15% vs.12%).^24^ Finally, UNICEF has provided pooled analyses from 25 LMICs indicating that perceived exposure to disability discrimination in the past 12 months was higher for adolescent girls with one or more functional limitations (19%) than among adolescent girls with no functional limitations (14%) and was more common among adolescents living in poorer than richer households.^25^

The omission of robust data on exposure to discrimination among adolescents is problematic given the critical importance of adolescence in providing a foundation for health and well-being in adulthood.^3^^,^^26–31^ The aims of our paper are to: (i) estimate the prevalence of self-reported exposure to multiple forms of discrimination among girls with/without disability and the relative risk of exposure to self-reported discrimination among girls with disability across a range of LMICs; (ii) estimate the relative risk of exposure to discrimination among girls with disability overall, and stratified by level of country human development; (iii) evaluate whether country level estimates of exposure to discrimination are associated with level of human development; (iv) identify potential risk factors for exposure to discrimination among girls with disability.

## Method

We undertook a secondary analysis of data collected in Round 6 (2017–2023) of UNICEF’s Multiple Indicator Cluster Surveys (MICS6).^32^^,^^33^ Specific details of the sampling procedures and ethical review and approval used in each country are available at http://mics.unicef.org/. All procedures conformed to the principles embodied in the Declaration of Helsinki. Our access to MICS data to undertake research on disability was approved by UNICEF. Given that we only had access to fully confidentialized data, no further ethical review was undertaken.

MICS6 contained several questionnaire modules. We used data from the household module, the module applied to all women aged 15–49 in the household and the module applied to a randomly selected child aged 5–17 from the household.^33^ All countries used cluster sampling methods to derive samples representative of the national population. All MICS questionnaires are translated into the local languages of each country and then back translated.

Inverse probability weights were supplied by country National Statistical Offices with technical support from UNICEF to ensure that responding participants were representative of the national/regional population. At the end of the download period (1 June 2025), nationally representative survey data containing both disability status and exposure to discrimination were available for 40 countries.

### Disability

MICS6 used the Washington Group Child Functioning Module (WG-CFM) to identify disability among adolescents. The module uses informant (typically maternal) report of child difficulties in 14 domains (see Table 3) Four response options were available for 11 domains [(1) ‘no difficulty’, (2) ‘some difficulty’, (3) ‘a lot of difficulty’, (4) ‘cannot do at all’]. The controlling behaviour domain had five response options with the reference point being other children of the same age [(1) ‘not at all’, (2) ‘less’, (3) ‘the same’, (4) ‘more’, or (5) ‘a lot more’]. The anxiety and depression domains had five response options [(1) ‘daily’, (2) ‘weekly’, (3) ‘monthly’, (4) ‘a few times a year’, (5) ‘never’].

During module development cognitive interviews were undertaken in six countries to ensure that questions were understood similarly in different languages and cultures.^34^ Validation of the module in three LMICs led to the recommendation of using a cut-off to define disability if the child had at least ‘a lot of difficulty’ in one or more domains or having ‘a lot more’ difficulty in controlling behaviour or showing signs of anxiety or depression ‘daily’.^35^ We used these cut-offs to define adolescent disability. Disability data were missing for < 1% of adolescents.

### Discrimination

Exposure to discrimination was based on responses to one question: ‘In the past 12 months, have you personally felt discriminated against or harassed on the basis of the following grounds? (1) Ethnic or immigration origin, (2) Sex, (3) Sexual orientation, (4) Age, (5) Religion or belief, (6) Disability, (7) For any other reason?’ Responses to this question were used to derive a binary variable (exposed to any form of discrimination in the past 12 months vs. not exposed). Discrimination data were missing for 1.5% of respondents with valid disability data.

### Country characteristics

We used the Human Development Index (HDI) for 2021 to characterize human development potential in participating countries. The HDI integrates three dimensions of human development: life expectancy at birth; mean and expected years of schooling; gross national income per capita.^36^^,^^37^ HDI data, which were available for all countries^38^ We used the HDI score to group countries into three levels of human development: high, medium and low.^38^

### Household wealth

MICS includes a within-country wealth index for each household based on the ownership of consumer goods, dwelling characteristics, water and sanitation, and other aspects of household wealth.^39–42^ Data were collected in all countries with no missing data.

### Highest level of education

The highest level of education of each woman in the household was recorded using country-specific categories. We recoded these data into a three-category measure: (i) no or pre-primary education; (ii) primary education; (iii) secondary or higher-level education. Data were collected in all countries and were missing for < 0.1% of respondents with valid disability data.

### Urban/rural location

Data were released with a within-country defined binary indicator of urban/rural location for each household. Data were missing for one country (Belarus). No data were missing in other countries.

### Approach to analysis

MICS guidelines suggest that results from samples of less than 25 children should be supressed. Given this, we excluded from all country-level analyses countries in which less than 25 adolescents had a disability. In addition, we excluded from all analyses three countries (Turks & Caicos Islands, Nauru, and Qatar) in which data was collected from fewer than 25 adolescent girls. Country-level analyses were undertaken in 25 countries, including data from 27 295 adolescent girls aged 15–17. Pooled analyses included 37 countries, including 30 705 adolescent girls.

In the first stage of analysis, we used descriptive statistics to derive country-level estimates of the prevalence of disability and exposure to discrimination.

In the second stage, we used descriptive statistics to estimate within country-level prevalence rates for adolescent girls with/without disability being exposed to discrimination. We also used Poisson regression with robust standard errors to estimate unadjusted prevalence rate ratios (PRR) for adolescent girls with disability being exposed to discrimination (adolescent girls without disability being the reference group).

In the third stage, we used mixed effects multilevel modelling (with random effects specified to allow association between disability and exposure to discrimination to vary across countries) to generate pooled estimates of: (i) the risk of adolescent girls with disability being exposed to discrimination (overall and stratified by HDI classification); (ii) the extent to which risk of exposure to discrimination among adolescent girls with disability varied by within country demographic characteristics (relative household wealth, highest level of maternal education and urban/rural location); and (iii) the risk of adolescent girls with specific functional limitations associated with being exposed to discrimination. In all analyses adolescent girls without disability were the reference group.

In the final stage, we used Spearman’s non-parametric correlation to investigate the strength of association between the HDI index and country-level estimates of exposure to discrimination (overall and for adolescent girls with/without disability).

All analyses were undertaken using Stata 16.1 using the Survey Data Analysis commands to take account of the clustering of observations by country and within country sampling clusters. All estimates included 95% confidence intervals (CI). Mixed effects multilevel modelling was undertaken using the mepoisson command with integration using mean–variance adaptive Gauss-Hermite quadrature to generate PRRs. UNICEF’s country-specific weights were used to take account of biases in sampling frames and household and individual level non-response. These weights were adjusted to ensure that the weighted sample sizes reflected identical sampling fractions of the under 18 population of children in 2021, as estimated and reported by UNICEF.^43^ Given the small amount of missing data, only participants with complete information were included in the analysis.

## Results

Details of the surveys are presented in Supplementary Table S1. The estimated prevalence of exposure to any form of discrimination among adolescent girls with/without disability, and the relative risk of exposure to any form of discrimination among girls with disability by country, are presented in Table 1. Across the 25 countries, adolescent girls with disability were more likely to experience discrimination than their peers in 22 countries—significantly so in eight countries. Adolescent girls with disability were less likely to experience discrimination than their peers in three countries, significantly so in one.

**Table 1:** Estimated exposure of adolescent girls with/without disability to any form of discrimination in 25 countries.

	With disability		No disability		Relative risk (reference group = adolescent girls without disability)
Country	Point estimate	95% CI	Point estimate	95% CI	
Trinidad & Tobago	32.0%	14.0–57.6	25.5%	17.9–35.0	1.25 (0.57–2.79)
Dominican Republic	22.9%	14.4–34.3	14.1%	11.3–17.4	1.63^*^ (1.00–2.64)
Cuba	0.3%	0.0–2.5	1.8%	0.5–6.6	0.19 (0.02–2.14)
Mongolia	6.2%	1.4–24.4	10.2%	6.7–15.2	0.61 (0.13–2.92)
Azerbaijan	14.5%	6.7–28.7	8.7%	5.8–12.8	1.67 (0.71–3.90)
Tunisia	21.7%	11.6–36.8	9.8%	7.4–12.9	2.29^***^ (1.54–3.41)
Uzbekistan	23.5%	9.2–48.1	4.8%	2.2–10.4	4.90^**^ (1.50–15.88)
Palestine	16.4%	7.1–33.4	11.9%	8.1–17.1	1.38 (0.56–3.37)
Guyana	22.0%	6.7–52.6	14.5%	9.8–20.8	1.52 (0.51–4.48)
Samoa	2.3%	0.5–9.4	10.9%	5.8–19.4	0.21^*^ (0.04–0.97)
Iraq	20.3%	11.2–34.1	11.0%	8.5–14.2	1.84 (0.98–3.48)
Bangladesh	17.5%	12.5–24.0	14.4%	13.1–15.8	1.22 (0.87–1.71)
Kiribati	40.3%	21.6–62.3	30.1%	21.7–40.1	1.34 (0.72–2.48)
Honduras	21.5%	14.6–30.4	11.1%	8.8–14.0	1.95^**^ (1.26–2.94)
Eswatini	19.0%	4.6–53.4	13.1%	7.3–22.6	1.45 (0.34–6.10)
Zimbabwe	22.4%	11.5–39.1	14.7%	11.2–18.9	1.53 (0.77–3.02)
Comoros	20.1%	10.6–35.0	19.3%	14.1–25.7	1.05 (0.54–2.02)
Pakistan	17.2%	14.4–20.5	10.3%	9.4–11.4	1.66^***^ (1.36–2.03)
Benin	30.0%	20.8–41.0	10.8%	8.2–13.9	2.79^***^ (1.82–4.27)
Lesotho	24.9%	12.8–42.6	17.4%	12.7–23.3	1.43 (0.75–2.72)
Malawi	31.5%	22.7–41.9	16.2%	13.1–19.9	1.94^***^ (1.35–2.79)
Madagascar	27.5%	18.9–38.2	15.6%	12.7–18.9	1.77^**^ (1.18–2.65)
DR Congo	29.5%	18.1–44.2	22.3%	16.9–28.8	1.32 (0.75–2.31)
Central African Republic	41.1%	30.1–52.9	35.1%	28.0–42.9	1.17 (0.82–1.68)
Chad	18.7%	12.6–26.9	16.3%	12.4–21.1	1.15 (0.72–1.84)

Notes: ^*^P < .05, ^**^P < .01, ^***^P < .001.

Results of mixed-effects multilevel modelling indicated that adolescent girls with disability were 1.66 times more likely to be exposed to any form of discrimination than adolescent girls without disability (PRR = 1.66 [1.40–1.96], *P* < .001). Similar associations were observed in countries with high human development (PRR = 1.94 [1.35–2.78], *P* < .001), medium human development (PRR = 1.56 [1.23–1.97], *P* < .001) and low human development (PRR = 1.55 [1.28–1.87], *P* < .001).

The estimated prevalence and relative risk of exposure to specific forms of discrimination among adolescent girls with/without disability are presented in Table 2. Overall and for each level of human development adolescent girls with disability were at higher risk than their peers of exposure to all forms of discrimination. Usually, these differences were statistically significant. Of the girls with disability who reported discrimination in the last year, only a minority (38%) reported exposure to disability discrimination. Among girls with disability who reported discrimination, but not disability discrimination: 39% reported discrimination based on their age; 37% based on their gender; 30% based on their ethnicity; 29% based on their religion; and 25% based on their sexual orientation.

**Table 2:** Estimated exposure of adolescent girls with/without disability to specific forms of discrimination.

	Form of discrimination						
	Disability	Ethnic or immigration origin	Sex	Sexual orientation	Age	Religion or belief	For any other reason
All countries							
With disability	4.0% (2.2–7.3)	6.0% (4.5–8.0)	7.7% (5.4–10.9)	4.2% (3.3–5.4)	8.5% (6.4–11.3)	5.9% (4.3–8.1)	8.6% (6.1–12.0)
Without disability	1.1% (0.9–1.4)	3.2% (2.6–3.9)	3.7% (3.3–4.2)	3.2% (2.8–3.7)	4.7% (4.0–5.5)	4.0% (2.9–5.4)	3.3% (2.8–4.0)
Risk	1.90^***^ (1.33–2.70)	1.58^***^ (1.23–2.02)	1.88^***^ (1.46–2.42)	1.43 (0.93–2.21)	1.51^**^ (1.11–2.06)	1.44^*^ (1.04–2.00)	1.87^***^ (1.33–2.63)
High human development							
With disability	0.8% (0.4–1.5)	3.7% (1.8–7.7)	4.9% (2.7–8.6)	0.8% (0.3–1.8)	9.5% (4.2–20.2)	1.3% (0.7–2.5)	5.5% (2.6–11.4)
Without disability	0.3% (0.2–0.5)	1.6% (1.1–2.4)	1.9% (1.4–2.7)	0.3% (0.2–0.5)	2.0% (1.3–3.0)	0.7% (0.5–1.0)	1.3% (1.0–1.8)
Risk	3.08^***^ (1.67–5.73)	1.89^***^ (1.38–2.58)	2.28^***^ (1.69–3.08)	1.77^**^ (1.23–2.55)	2.51^*^ (1.14–5.49)	1.49 (0.73–3.07)	3.06^***^ (1.66–5.64)
Medium human development							
With disability	4.2% (2.6–6.7)	6.7% (2.6–16.3)	10.7% (5.8–18.9)	6.8% (4.5–10.0)	5.5% (3.7–8.0)	3.3% (2.0–5.4)	3.6% (2.1–6.1)
Without disability	0.7% (0.5–1.1)	2.0% (1.6–2.5)	4.4% (3.6–5.3)	6.3% (5.4–7.3)	4.6% (3.9–5.5)	1.9% (1.5–2.4)	1.1% (0.8–1.6)
Risk	2.58^***^ (1.64–4.07)	2.01 (0.93–4.34)	1.94^**^ (1.19–3.17)	1.24 (0.91–1.70)	1.22^*^ (1.05–1.43)	1.46 (0.91–2.33)	2.42^***^ (1.54–3.80)
Low human development							
With disability	4.3% (2.1–8.9)	6.1% (4.5–8.1)	7.3% (4.6–11.4)	4.3% (3.2–5.6)	9.1% (6.4–12.7)	6.7% (4.8–9.3)	10.1% (6.8–14.6)
Without disability	1.5% (1.2–1.9)	3.9% (3.1–5.0)	3.9% (3.3–4.6)	3.0% (2.5–3.7)	5.3% (4.3–6.5)	5.3% (3.8–7.4)	4.6% (3.8–5.6)
Risk	1.61 (1.00–2.61)	1.42^*^ (1.07–1.89)	1.85^**^ (1.24–2.75)	1.44 (0.78–2.67)	1.43 (0.95–2.15)	1.49 (0.93–2.38)	1.63^*^ (1.02–2.61)

Notes: ^*^P < .05, ^**^P < .01, ^***^P < .001.

The association between type of functional limitation and exposure to discrimination (any discrimination and disability-related discrimination) is presented in Table 3. Adolescent girls with disability were at increased risk of exposure to discrimination and disability related-discrimination compared to adolescent girls without disability for all forms of functional limitation. These differences were all statistically significant for exposure to any form of discrimination. While increased risk of exposure to disability discrimination was higher for all limitations (when compared to no disability) these differences were only statistically significant for 5 of the 13 limitations measured.

**Table 3:** Pooled analyses of risk of discrimination by specific functional limitation associated with disability.

	Discrimination (any)	Disability discrimination
		
Self-care	3.31^***^ (2.16–5.06)	2.12 (0.68–6.63)
Walking	3.07^***^ (2.16–4.34)	2.99 (0.52–17.07)
Hearing	2.98^***^ (1.80–4.94)	1.10 (0.46–2.64)
Speech	2.92^*^ (1.29–6.61)	1.53 (0.11–22.30)
Concentrating	2.77^***^ (1.68–4.54)	4.98^***^ (2.24–11.04)
Remembering	2.52^***^ (1.92–3.30)	2.54^*^ (1.01–6.39)
Controlling behaviour	2.35^***^ (1.64–3.38)	1.52 (0.50–4.68)
Learning	2.34^***^ (1.53–3.58)	1.36 (0.46–4.03)
Making friends	2.32^***^ (1.69–3.20)	2.17 (0.96–4.91)
Dealing with change	2.17^***^ (1.46–3.23)	2.72^*^ (1.16–6.38)
Seeing	1.69^*^ (1.04–2.74)	1.26 (0.63–2.52)
Depression	1.49^**^ (1.13–1.97)	1.83^**^ (1.26–2.67)
Anxiety	1.35^*^ (1.07–1.70)	1.53^*^ (1.02–2.31)

Notes: ^*^P < .05, ^**^P < .01, ^***^P < .001.

Analyses of the extent to which risk of exposure to discrimination among adolescent girls with disability varied by relative household wealth, highest level of maternal education and urban/rural location are presented in Table 4. While risk of exposure to discrimination was not significantly related to urban/rural location, it was significantly more likely among adolescent girls with disability living in poorer households and among girls whose mother did not have secondary level education. Multivariate analyses (Supplementary Table S2) indicated that risk of exposure to discrimination was primarily associated with level of maternal education.

**Table 4:** Bivariate associations between living arrangements and exposure to discrimination among adolescent girls with disability.

Relative household wealth		
	Richest quintile	1 (reference group)
	Quintile 2	0.83 (0.61–1.12)
	Quintile 3	1.41^*^ (1.07–1.87)
	Quintile 4	1.54^***^ (1.23–1.92)
	Poorest quintile	1.60^**^ (1.14–2.24)
Highest level of maternal education		
	Secondary	1 (reference group)
	Primary	1.67^***^ (1.37–2.02)
	None/pre-primary	1.86^***^ (1.50–2.30)
Location		
	Rural	1 (reference group)
	Urban	0.79 (0.60–1.04)

Notes: ^*^P < .05, ^**^P < .01, ^***^P < .001.

Across the 37 countries there were significant associations between country HDI score and the prevalence of disability (rho = −0.520 [−0.727 to −0.226], *P* < .001) and exposure to discrimination (rho = −0.514 [−0.723 to −0.219], *P* < .01). Across the 25 countries in which within country analyses were undertaken there was no significant association between country HDI score and the relative risk of adolescent girls with disability being exposed to discrimination (rho = −0.038 [−0.437 to +0.374], *P* > .05).

## Discussion

### Main finding of this study

Our analyses of the reported experiences of 30 705 adolescent girls aged 15–17 in 37 countries indicated that: (i) adolescent girls with disability were 1.66 times more likely to be exposed to discrimination than adolescent girls without disability; (ii) in 25 countries in which within-country analyses were undertaken, adolescent girls with disability were more likely to be exposed to discrimination in 22 (88%) countries, significantly so in 8 countries; (iii) adolescent girls with disability were at higher risk of exposure to all forms of discrimination measured; (iv) adolescent girls with disability were significantly more likely to be exposed to discrimination if they lived in poorer households and had mothers with lower than secondary level education; (v) while exposure to discrimination was significantly more common in countries with lower levels of human development, there was no association between level of human development and the magnitude of the risk of adolescent girls with disability being exposed to discrimination.

### What is already known on this topic

Little was known about the prevalence of exposure to discrimination among adolescent girls with/without disability, especially in LMICs.

### What this study adds

The main strength of our study is the use of 37 nationally representative surveys with high response rates undertaken in a wide range of countries. Our results add significantly to the existing literature in two ways. First, they suggest that in the majority of LMICs adolescent girls with disability are at significantly increased risk of exposure to discrimination than their peers. As noted above, exposure to discrimination represents a violation of the right of people with disability to participate in society on equal terms with others and increases their risk of exposure to well-established social determinants of poorer health.^3–11^ This is particularly problematic given the critical importance of adolescence in providing a foundation for health and well-being in adulthood.^3^^,^^26–31^

Our analyses extend those undertaken by UNICEF in six ways: (i) we included all forms of discrimination, rather than just disability discrimination; (ii) we included data from an additional 10 countries; (iii) we reported country level data for all countries with adequate sample sizes; (iv) we provided estimates of the risk of discrimination for 13 (rather than 6) functional limitations associated with disability and three additional contextual factors (relative household wealth, level of maternal education, urban/rural status); (v) we used an alternative approach for pooling data (mixed effects multilevel modelling); and (vi) we investigated whether risk of exposure was associated with a country-level measure of human development.

Second, while exposure to disability discrimination has received considerable attention, it is clear that adolescent girls are also at risk of exposure to discrimination on the basis of other potentially intersecting sources of marginalization (e.g. age, gender, ethnicity, sexual orientation, level of maternal education).^12^^,^^24^ Future research is needed to identify the extent and characteristics of these potentially intersecting sources of marginalization. It is notable that of the adolescent girls with disability who reported discrimination in the last year only a minority (38%) reported exposure to disability discrimination.

Future United Nations Disability and Development Reports should consider including data on the exposure of people with disability to non-disability related discrimination. These results also support the inclusion of indicators of discrimination and membership of one or more potentially marginalized groups (e.g. people with disability) in frameworks for monitoring health equity.^44^^,^^45^ Future research should focus on whether similar results are evident among adolescent boys and adolescents with mild disability.

### Limitations of this study

Four important limitations need to be considered when evaluating these results. First, limited information was collected on the nature of discrimination (e.g. no information was collected on the identity of perpetrators, location or number of times exposed). Second, disability was determined by caregiver report, rather than adolescent self-report. Third, the WG-CFM does not include any measure of the extent or forms of restrictions in participation that are associated with reported functional limitations. Finally, the use of the cut-off in the WG-CFM of at least ‘a lot of difficulty’ fails to identify participants with mild disability, including those with multiple forms of mild disability that may be associated with significant disability.^46^

## Supplementary Material

Supplementary_Tables_fdag054

## Data Availability

The data underlying this article are available on approval from UNICEF in their MICS data archive at https://mics.unicef.org/surveys.

## Notes

**Eric Emerson,** Emeritus Professor of Disability and Health Research, Honorary Professor, Honorary Professor of Disability

**Katrina Scior,** Professor of Clinical Psychology and Stigma Studies

**Gwynnyth Llewellyn,** Professor Emerita, Co-Head, WHO Collaborating Centre for Strengthening Rehabilitation Capacity in Health Systems, Honorary Professorial Fellow
