# Supplementary material for: The exposure of adolescent girls with/without disabilities to discrimination: cross-sectional analyses of nationally representative surveys undertaken in 37 countries
Source: J Public Health (Oxf). 2026 Jun 27;48(3):691–9. doi: 10.1093/pubmed/fdag054 (PMC13531698; doi:10.1093/pubmed/fdag054)
Supplement: Supplementary file 1 [file supplementary_tables_fdag054.docx]

| Supplementary Table S1: Survey details |  |  |  |  |  |  |
| --- | --- | --- | --- | --- | --- | --- |
| Country | Year of survey | HDI  (2021) | Response rates of randomly selected child aged 5-17 | Sample size (unweighted) | Prevalence of disability (with 95% CI) | Prevalence of exposure to any form of discrimination (with 95% CI) |
| *Very High Human Development* |  |  |  |  |  |  |
| Montenegro | 2018/19 | 0.832 | 60.5% | 92 | 6.8% (3.2-14.0) | 4.3% (1.7-10.5) |
| Trinidad & Tobago | 2022 | 0.810 | 88.3% | 254 | 10.3% (6.2-16.7) | 25.7% (18.7-34.3) |
| Belarus | 2019 | 0.808 | 95.6% | 174 | 2.4% (0.9-6.2) | 6.6% (2.9-14.3) |
| Georgia | 2018 | 0.802 | 83.4% | 228 | 8.8% (4.9-15.3) | 4.6% (2.0-10.4) |
| Serbia | 2019 | 0.802 | 80.8% | 131 | 1.6% (0.4-5.9) | 4.9% (2.2-10.7) |
| *High Human Development* |  |  |  |  |  |  |
| North Macedonia | 2018/19 | 0.770 | 86.8% | 126 | 11.0% (3.9-27.3) | 14.4% (7.7-25.1) |
| Dominican Republic | 2019 | 0.767 | 96.1% | 1,418 | 8.9% (7.1-11.2) | 14.8% (12.1-18.0) |
| Cuba | 2019 | 0.764 | 98.5% | 348 | 9.0% (5.5-14.5) | 1.6% (0.5-5.5) |
| Turkmenistan | 2019 | 0.745 | 97.1% | 292 | 2.3% (0.9-5.9) | 1.2% (0.4-3.3) |
| Tonga | 2019 | 0.745 | 96.1% | 180 | 8.7% (4.8-15.4) | 21.7% (13.8-32.4) |
| Mongolia | 2018 | 0.739 | 95.7% | 502 | 10.7% (6.9-16.1) | 9.9% (6.6-14.5) |
| Azerbaijan | 2023 | 0.738 | 93.4% | 471 | 7.6% (5.5-10.4) | 9.4% (6.6-13.3) |
| Tunisia | 2018/23 | 0.731 | 86.2% | 436 | 20.9% (18.3-23.7) | 12.4% (8.6-17.4) |
| Fiji | 2021 | 0.730 | 96.8% | 261 | 5.2% (3.0-8.9) | 13.6% (8.4-20.9) |
| Uzbekistan | 2021 | 0.727 | 99.6% | 232 | 14.5% (9.3-22.1) | 7.3% (4.0-13.0) |
| Palestine | 2019/20 | 0.715 | 94.0% | 505 | 9.9% (7.2-13.5) | 12.3% (8.8-17.0) |
| Guyana | 2019/20 | 0.714 | 89.3% | 331 | 19.7% (12.3-30.1) | 15.8% (10.0-24.0) |
| Jamaica | 2022 | 0.709 | 93.3% | 293 | 5.6% (3.3-9.3) | 13.9% (10.2-18.7) |
| Samoa | 2019/20 | 0.707 | 93.0% | 184 | 22.9% (16.2-31.4) | 8.9% (4.9-15.5) |
| Viet Nam | 2020 | 0.703 | 97.3% | 594 | 0.9% (0.4-2.0) | 3.9% (2.4-6.3) |
| *Medium Human Development* |  |  |  |  |  |  |
| Kyrgyzstan | 2018 | 0.692 | 97.4% | 292 | 5.8% (3.6-9.0) | 3.6% (2.0-6.4) |
| Iraq | 2018 | 0.686 | 99.4% | 1,458 | 16.7% (13.7-20.2) | 12.5% (9.9-15.8) |
| Bangladesh | 2019 | 0.661 | 96.4% | 3,997 | 5.3% (4.5-6.2) | 14,5% (13.3-15.9) |
| Tuvalu | 2019/20 | 0.641 | 96.8% | 31 | 3.3% (1.3-8.4) | 12.6% (6.3-23.9) |
| Kiribati | 2018/19 | 0.624 | 98.2% | 177 | 17.3% (11.4-25.5) | 31.9% (24.1-40.8) |
| Honduras | 2019 | 0.621 | 90.4% | 1,203 | 17.5% (14.9-20.4) | 13.1% (10.7-15.9) |
| Eswatini | 2021 | 0.597 | 93.3% | 2,490 | 11.0% (6.9-17.2) | 13.7% (8.1-22.3) |
| Zimbabwe | 2018/19 | 0.593 | 96.3% | 566 | 8.6% (6.1-11.9) | 15.3% (12.0-19.4) |
| Comoros | 2022 | 0.558 | 95.2% | 404 | 20.2% (15.5-25.8) | 20.0% (15.2-25.8) |
| *Low Human Development* |  |  |  |  |  |  |
| Pakistan | 2017-19 | 0.544 | 93.8% | 7,803 | 12.4% (11.4-13.5) | 11.1% (10.2-12.1) |
| Benin | 2021 | 0.525 | 98.7% | 932 | 11.9% (9.3-15.2) | 13.2% (10.5-16.4) |
| Lesotho | 2018 | 0.514 | 90.1% | 426 | 11.9% (8.6-16.3) | 18.4% (13.9-24.0) |
| Malawi | 2019/20 | 0.512 | 97.9% | 1,469 | 10.6% (8.6-13.0) | 17.9% (14.8-21.4) |
| Madagascar | 2018 | 0.501 | 94.1% | 1,071 | 15.1% (12.2-18.6) | 17.3% (14.5-20.6) |
| DR Congo | 2017/18 | 0.479 | 99.9% | 1,170 | 14.0% (10.7-18.1) | 23.6% (18.8-29.2) |
| Central African Republic | 2018/19 | 0.404 | 96.3% | 429 | 30.1% (24.6-36.3) | 37.1% (31.0-43.6) |
| Chad | 2019 | 0.394 | 99.4% | 1,192 | 28.1% (23.8-32.9) | 17.2% (13.9-21.2) |

| Supplementary Table S2: Multivariate Predictors of Exposure to Discrimination Among Adolescent Girls with Disability | |
| --- | --- |
|  |  |
| Relative Household Wealth | APPR |
| Richest quintile | 1.0 (ref) |
| Quintile 2 | 0.67* (0.49-0.91) |
| Quintile 3 | 0.92 (0.68-1.25) |
| Quintile 4 | 0.90 (0.73-1.11) |
| Poorest quintile | 0.88 (0.58-1.32) |
| Highest Level of Maternal Education |  |
| Secondary | 1.0 (ref) |
| Primary | 1.58*** (1.34-1.87) |
| None/pre-primary | 1.74*** (1.44-2.11) |
| Location |  |
| Rural | 1 (reference group) |
| Urban | 0.81 (0.60-1.11) |
| Notes: * p<0.05, ** p<0.01, *** p<0.001 |  |
